# Reference High-Voltage Sensing Chain for the Assessment of Class 0.1-WB3 Instrument Transformers in the Frequency Range up to 150 kHz According to IEC 61869

**DOI:** 10.3390/s25206416

**Published:** 2025-10-17

**Authors:** Mohamed Agazar, Claudio Iodice, Mario Luiso

**Affiliations:** 1 Laboratoire National de Métrologie et d’Essais (LNE), 1 Rue Gaston Boissier, 75015 Paris, France; 2 Department of Engineering, Università degli Studi della Campania “Luigi Vanvitelli”, Via Roma 29, 81031 Aversa, Italy; claudio.iodice@unicampania.it (C.I.); mario.luiso@unicampania.it (M.L.)

**Keywords:** instrument transformers, supraharmonics (9–150 kHz), high voltage divider, power quality, medium voltage, measurement uncertainty

## Abstract

This paper presents the development and characterization of a reference high-voltage sensing chain for the calibration and conformity assessment of instrument transformers with Class 0.1-WB3, in the extended frequency range up to 150 kHz, according to IEC 61869. The sensing chain, composed of a high-voltage divider, precision attenuators and high-pass filters, has been specifically developed and characterized. The chain features two parallel measurement paths: the first path, comprising the high-voltage divider and attenuator, is optimized for measuring the fundamental frequency superimposed with high-amplitude harmonics; the second path, consisting of the high-voltage divider followed by a high-pass filter, is dedicated to measuring very-low-level superimposed harmonic components by enhancing the signal-to-noise ratio. These two paths are integrated with a digitizer to form a complete and modular measurement chain. The expanded uncertainty of measurement has been thoroughly evaluated and confirms the chain’s ability to support assessment of instrument transformers with Class 0.1-WB3 compliance. Additionally, the chain architecture enables a future extension up to 500 kHz, addressing the growing need to evaluate instrument transformers under high-frequency power quality disturbances and improving the sensing capability in this field.

## 1. Introduction

The ongoing transformation of electrical grids toward decentralized and converter-based operation has introduced complex waveform distortions extending far beyond the traditional harmonic range. Modern power electronic devices such as photovoltaic inverters, variable speed drives and switched-mode power supplies generate distortion components up to 150 kHz, commonly referred to as supraharmonics [1,2,3]. These components can propagate through low- and medium-voltage grids potentially interfering with control, protection and metering systems [4,5,6]. Measurement and analysis techniques for supraharmonics at low voltage are well documented in standards such as IEC 61000-4-7 and IEC 61000-4-30 [7,8] and in numerous studies [9,10,11].

At medium voltage, instrument transformers (ITs), including conventional inductive transformers, low-power instrument transformers (LPITs), resistive or capacitive dividers and hybrid sensors, serve as indispensable interfaces between the power system and measurement equipment. Their primary function is to scale down voltage or current while preserving waveform integrity. The wide variety of IT technologies leads to diverse frequency responses and can introduce amplitude and phase deviations that increase significantly above the power frequency [12]. These effects distort the measurement of emitted disturbances and compromise the reliability of power quality (PQ) assessments. Therefore, developing traceable calibration methods capable of characterizing ITs up to 150 kHz is essential to ensure measurement comparability and support compliance with international standards.

Recognizing this need, the IEC 61869-1:2023 (Edition 2) standard introduced optional wideband (WB) accuracy classes for ITs: WB0 (up to the 13th harmonic), WB1 (up to 3 kHz), WB2 (up to 20 kHz), WB3 (up to 150 kHz) and WB4 (up to 500 kHz) [13]. These classes, which can be appended to a base accuracy class (e.g., Class 0.1-WB3), specify permissible magnitude and phase errors over defined frequency intervals. This approach, consistent with IEC 61869-6 [14] for LPITs, provides a unified framework for performance specification in the supraharmonic range. However, the standard does not prescribe calibration procedures, leaving open the question of how to establish traceable measurement systems. In this context, metrological traceability and associated measurement uncertainty are critical to ensuring the validity of conformity assessments. Metrological traceability refers to the property of a measurement result whereby it can be related to a reference, typically an SI unit, through an unbroken and documented chain of calibrations, each contributing to the overall uncertainty [15].

In recent years, significant progress has been achieved worldwide toward extending calibration capabilities for ITs into the wideband domain. The EMPIR 19NRM05 IT4PQ project [16] developed reference methods and calibration chains [17,18,19] for ITs up to 9 kHz, laying the foundation for subsequent work. Stano et al. [20] experimentally evaluated the IEC 61869-1 WB1 accuracy classes for inductive current transformers, while Giordano et al. [21] proposed a medium-voltage setup for wideband characterization of voltage transformers up to 150 kHz. Beyond Europe, National Metrology Institutes (NMIs) and universities [22,23,24] have developed reference systems extending up to 100 kHz. Yan et al. [25] presented a reference system for high-voltage transducers up to 10 kHz and Rom et al. [26] demonstrated a wideband-power-analyzer-based calibration of current transformers up to 150 kHz. Together, these works confirm that extending calibration into the supraharmonic range is both feasible and increasingly necessary.

However, most of these efforts do not specifically address the calibration and conformity assessment of ITs with respect to the IEC 61869 wideband accuracy classes, particularly WB2 and WB3. They also do not adequately address the measurement of harmonics with very low amplitudes. In practice, supraharmonic components exhibit amplitudes several orders of magnitude lower than the fundamental, requiring measurement systems with exceptionally high dynamic ranges. Meeting the standard limits for weak harmonics, down to 0.1% or even 0.01% of the fundamental, demands reference systems capable of resolving these low-level components without distortion or noise coupling from the fundamental. Yet, most existing approaches rely either on simplified methods performing frequency-response measurements at low amplitudes or on reference methods combining fundamental and harmonic components but limited to lower frequency ranges and high amplitudes of harmonics (e.g., ≥1% of the fundamental). A reference method extending up to 150 kHz, particularly for voltage transformers, is still lacking. This gap represents one of the key technical barriers to achieving reliable, standard-based compliance for medium-voltage ITs in the supraharmonic range.

To address these limitations, this work focuses on developing a new reference chain specifically designed to assess ITs with the most demanding accuracy requirements. This development is one of the objectives of the ADMIT project [27]. The chain supports the conformity assessment of ITs with a metrological class of 0.1, as well as Class WB3, as defined in IEC 61869, which specifies maximum errors of 0.1% at the fundamental frequency, 1% up to 20 kHz, 2% up to 50 kHz and 5% up to 150 kHz. To reliably assess such devices, the target expanded uncertainty of the measurement chain should be at least five times lower than these limits, namely, 0.02% at the fundamental, 0.2% up to 20 kHz and 1% up to 150 kHz. Achieving such low uncertainties under high-voltage and high-frequency conditions requires a complete rethinking of the measurement architecture. This includes the design of a new voltage divider with flat amplitude and phase response capable of resolving small harmonic components superimposed on large fundamental voltages, as well as the integration of dedicated attenuators, filters and acquisition chains.

## 2. Calibration of Voltage Transformers up to 150 kHz

The calibration methodology described in this section establishes the reference chain for assessing voltage transformers in the extended frequency range up to 150 kHz. It provides the practical implementation of the proposed approach. This section first presents the general and theoretical principles of calibration (Section 2.1), then details the adopted chain architecture (Section 2.2) and is followed by the design and characterization of its key components in Section 3.

### 2.1. Calibration Principle

**Figure 1:**
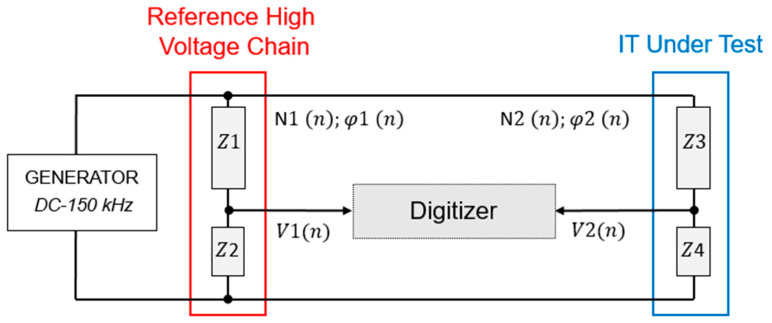
Assessment of amplitude and phase of an instrument transformer by comparison to a reference high-voltage chain and a digitizer.

The assessment of the amplitude and phase of ITs is carried out using a reference high-voltage chain (RHVC) and a digitizer, as presented in Figure 1. The RHVC is necessary to scale down the high voltage to levels manageable by the digitizer. A signal generator produces the excitation signal covering the full frequency range of interest up to 150 kHz. The generator is similar to those developed in [16] but has been extended to up to 150 kHz and will be subject to a separate publication. This signal represents a test voltage simulating either fundamental or harmonic components. The RHVC (left side) is composed of impedances Z1 and Z2 with known characteristics denoted N1(n);φ1(n), where *n* is the harmonic order, N1(n) is the amplitude transfer function (scale factor) and φ1(n) is the phase response. The IT under test (right side) is composed of impedances Z3 and Z4. Its unknown scale factor and phase response are N2(n) and φ2(n), respectively. Each output is connected to a digitizer, acquiring $V1\left(n\right)$, the output voltage of the RHVC and $V2\left(n\right)$, the output voltage of the IT. To determine the frequency dependent of the scale factor, Equation (1) is used. The phase response of the IT is calculated using Equation (2).

(1) $$N2\left(n\right)=N1\left(n\right)\cdot \frac{V1\left(n\right)}{V2\left(n\right)}=N1(n)\cdot N3(n)$$

(2) $$\varphi 2\left(n\right)=\varphi 1\left(n\right)+∆\varphi (n)$$

where  N3(n) and ∆φ(n) are the ratio and phase difference measured between the amplitudes V1(n) and  V2(n) using the digitizer.

The calibration of the IT depends on the scale factor and phase response of the high-voltage divider (HVD), as well as on the measured voltage ratio and phase difference between the output voltages in the digitizer side. The traceability and uncertainty of the calibration mainly rely on this equipment (HVD + Digitizer).

### 2.2. RHVC Principle

The RHVC acts as a calibrated sensor front-end that converts high-voltage signals into measurable low-voltage quantities with preserved amplitude and phase information. It should be capable of operating under both DC and AC conditions depending on the type of the IT. For DC applications, test voltages can reach up to 50 kV and measurements are typically performed at 100% of the rated voltage. For AC applications, the reference condition is usually a 50 Hz (or 60 Hz) sinusoidal waveform with a test voltage of around 35 kV rms (50 kVp), also applied at nominal voltage. Harmonic components are intentionally superimposed on the fundamental waveform. These harmonics can have amplitudes ranging from 0.01% to 1% of the fundamental voltage. The key challenge lies in accurately detecting and quantifying such small harmonic components, both in amplitude and phase, when they are superimposed on a large fundamental voltage. This becomes even more demanding when the frequency of the harmonic content extends up to 150 kHz. The chain must therefore ensure excellent linearity, high dynamic range and minimal phase error across a wide bandwidth.

To address this, two main measurement approaches can be considered. The first technique involves using a single high-voltage divider (HVD) connected to a digitizer with a very high dynamic range. This allows the chain to simultaneously capture both the high-amplitude fundamental component and the much smaller harmonic components. The main advantage of this approach is that it preserves the full waveform and avoids the need for any signal conditioning. However, the drawback lies in the difficulty of finding digitizers or acquisition chains that offer sufficiently high resolution, linearity and noise performance to resolve very-low-level signals (e.g., 0.01% of the fundamental).

The second approach consists of applying filtering techniques to suppress the fundamental frequency and retain only the harmonic components. This can significantly reduce the dynamic range requirement of the acquisition chain, as the large fundamental component is no longer present in the captured signal. However, this method introduces its own challenges. The accuracy of the measurement can be affected by the non-linearity and imperfections of the filter, particularly in terms of amplitude flatness and phase response.

**Figure 2:**
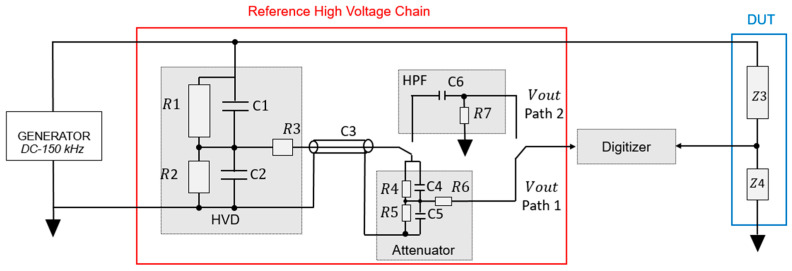
Overview schematic of the reference measurement chain for instrument transformer calibration up to 150 kHz. The chain includes the generator; the reference high-voltage chain (RHVC) with the high-voltage divider (HVD), attenuator and high-pass filter; and the device under test (DUT). Two measurement paths are available: Path 1 (HVD + attenuator) for fundamental and higher-amplitude harmonics and Path 2 (HVD + high-pass filter) for low-level harmonic components. Both paths are connected to the digitizer for synchronous acquisition.

Each technique has its own advantages and limitations and the choice between them depends on the specific performance targets and available instrumentation. In our case, the target is to be able to assess Class 0.1-WB3 ITs with a target uncertainty five times lower than the accuracy limits defined in IEC 61869-1, namely, 0.02% for the fundamental frequency, 0.2% up to 20 kHz and 1% up to 150 kHz. To overcome the limitations of each individual technique, we have adopted a dual-path architecture that implements both approaches, enabling redundancy, flexibility and improved reliability in harmonic measurements. This hybrid strategy allows for cross-verification. The implemented chain is shown in Figure 2.

The RHVC begins with the HVD composed of resistive and capacitive elements (RC structure). The scale factor of the divider has been chosen to be 500. This scaling ensures that the harmonic signal remains high enough for the filtration operation. On the other hand, the fundamental component, after division, reaches a level of ±100 Vp at the output of the HVD. To bring this signal within the input range of most digitizers, an attenuation stage is developed to reduce the signal to ±1 Vp, compatible with both the input range and linearity specifications of most high-performance digitizers.

The selection between Path 1 and Path 2 is based on the signal amplitude at the digitizer input. Path 1 (HVD + attenuator) is used whenever the expected input signal is higher than approximately 1 mV (this limitation is defined according to the characterization results of the selected digitizer, see Section 4), which guarantees an adequate signal-to-noise ratio. Otherwise, Path 2 (HVD + high-pass filter + amplifier) is employed to enhance sensitivity. As an illustration, for a fundamental of 50 kV, a harmonic at 0.1% of the fundamental corresponds to 50 V at the primary and about 1 mV at the digitizer input after division (scale factor 50,000), making Path 1 appropriate. However, for lower fundamental voltages, the same harmonic ratio results in signals below 1 mV, and Path 2 must therefore be used. This criterion ensures robust operation across different primary voltage levels.

## 3. Design of the RHVC

### 3.1. Design of the HVD

The voltage divider is the primary sensing element that scales down the voltage. The development of a new voltage divider is necessary to overcome the limitations of currently available commercial dividers and existing in-house solutions. Most of these dividers are specifically designed for DC, AC@50–60 Hz or impulse voltage measurements and are not optimized for broadband applications involving harmonic content up to 150 kHz. They often feature large scale factors (e.g., 10,000), which limit the output signal amplitude and reduce the visibility of low-level harmonic components, especially when their amplitude represents very small fraction of the fundamental waveform. In addition, these dividers typically suffer from higher noise-to-signal ratios and limited or non-linear frequency responses, making them unsuitable for precise amplitude and phase characterization of weak harmonic signals superimposed on the fundamental.

The theoretical framework and background of HV voltage dividers are discussed in our paper [28]. We have decided to choose an RC structure which combines resistive and capacitive branches in parallel and which, with proper compensation, can reach a bandwidth of several MHz. The high-voltage arm consists of a resistor of *R*1 = 100 MΩ and a capacitor of *C*1 = 100 pF. Moreover, to maintain waveform fidelity, the time constant of the low-voltage part (comprising R2, C2, R3 and C3, as well as the downstream attenuator components R4, R5, C4 and C5) has been precisely adjusted to match the time constant of the high-voltage arm (R1, C1). This compensation ensures that the divider maintains a flat frequency response and minimal phase shift across the operating bandwidth.

**Figure 3:**
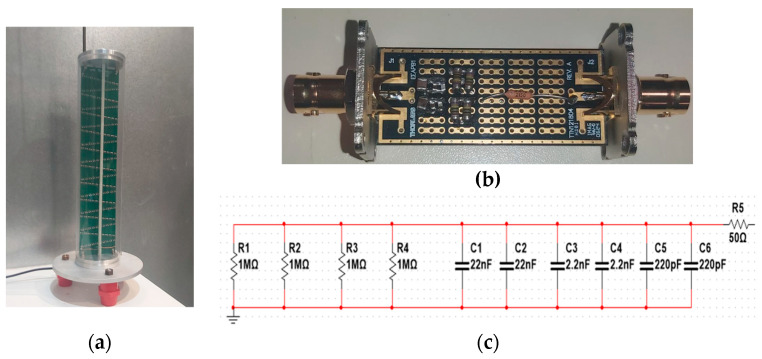
The developed RC high-voltage divider for voltages up to 50 kVp: (**a**) high-voltage arm using NP0 capacitors and metallic resistors on a PCB with a zigzag structure; two PCBs mounted in parallel and in opposite directions to reduce electric field influence. (**b**) Low-voltage arm with a compact coaxial structure, using the same SMD components as the high-voltage arm; (**c**) electrical circuit of the low-voltage arm.

The HVD that was developed is shown in Figure 3. To build the resistor R1, we have selected resistors of 1 MΩ ± 0.1% from Vishay, type MELF (metal electrode leadless face), 400 mW, 350 V and 15 ppm/°C, due to their excellent voltage linearity. We have measured their voltage linearity from 10 V to 350 V, and they exhibited very good performance, with a voltage deviation better than 0.005%. When the rated voltage was applied continuously for 10 min, the resistors showed minimal self-heating effects with a deviation better than 0.001%, confirming excellent thermal stability under power dissipation. We have developed a 50 kV (+20% of safety margin) section by assembling 200 resistors in series using a zigzag structure on a printed circuit board (PCB). Despite the excellent characteristics of each individual component, applying high voltage has generated intense electric fields around the resistors which have affected the voltage linearity by 0.1%. To mitigate the influence of these electric fields, we have decided to use two PCBs placed in parallel and opposite directions, so the current flowing though one board circulates in the opposite direction of the second one. This configuration significantly improves the overall voltage linearity. The value of R1 in this case is about 100 MΩ (two 200 MΩ in parallel) and the DC current at the rated voltage is equal to 0.5 mA at 50 kV, which is high enough to eliminate the influence of the leakage current.

To build the capacitor C1, only one type of capacitor meets our requirements: negative/positive-zero (NP0) ceramic capacitors, known for their very low temperature coefficient (TC), typically less than 30 ppm/°C. Polypropylene capacitors also offer good performance but their higher TC (e.g., <300 ppm/°C) exceeds the target uncertainty of 0.02% at the fundamental frequency. The value of C1 has been selected to be 100 pF to minimize the influence of parasitic capacitance to ground and to limit power dissipation from the voltage source. Murata capacitors rated at 10 nF and 650 Vpp have been chosen. Each capacitor has been soldered in parallel with each MELF resistor.

In the low-voltage arm of the HVD, the same component technologies as those used in the high-voltage arm have been employed. This approach helps cancel out mutual influences such as temperature, voltage and frequency behaviors. Moreover, the low-voltage arm has been designed with a compact layout to better meet these conditions and minimize parasitic effects. The components R2 and C2 have been soldered in a coaxial configuration to eliminate the influence of stray capacitance to ground. R3 is a damping resistor and its value is equal to the characteristic impedance of the coaxial cable (50 Ω).

The HVD has been calibrated under various conditions to characterize its scale factor and dynamic behavior. The main results are presented in Table 1, and the output of the divider has been loaded using a measuring instrument with an input impedance equivalent to that of the attenuator (see below).

At AC@50 Hz, the divider was calibrated from 1 kV to 50 kV yielding a scale factor of 500.848 with a relative deviation of 0.004%. A second calibration at AC@50 Hz, from 1 kV to 35 kV rms, produced a scale factor of 500.729 with a deviation of 0.011%. Additionally, a frequency sweep from DC to 150 kHz was performed at 200 V, resulting in a scale factor of 500.37 with a maximum deviation of 0.10%. Regarding phase behavior, a time constant of −38 ns was measured, characterizing the phase shift performance of the divider across frequency, and can be calculated using Equation (3):

(3) Phase (rad)=2·π·f·t

where f is the frequency of the signal and t is the time constant.

The calibration results demonstrate that the HVD offers excellent performance in terms of accuracy and stability across a wide range of voltages and frequencies. The divider meets the stringent accuracy requirement in terms of the scale factor from DC to 150 kHz. Additionally, the measured time constant of −38 ns ensures reliable correction of the phase behavior for each frequency.

### 3.2. Design of the Attenuator

The attenuator plays a crucial role in the measurement chain by further reducing the already scaled-down voltage from the HVD to a level compatible with the input range of the measurement instrument such as a digitizer. Beyond voltage reduction, the attenuator also aids in impedance matching between the divider and the measuring device, minimizing signal reflections and preserving measurement accuracy. By providing a stable and linear attenuation over a wide frequency range, it helps maintain signal integrity. The input impedance of the attenuator is designed to emulate the input impedance of the digitizer; therefore, when the attenuator is connected, HVD frequency behavior does not change if compared to the behavior measured with HVD only. As, for the case at hand, the input impedance of the digitizer is typically about 1 MΩ//30 pF, while the input impedance of the attenuator is fixed to R4 ≅ 1 MΩ and C4 ≅ 30 pF. Further pieces of information about the digitizer will be provided in Section 4.

The developed attenuator has a scale factor of 100. It is built using components similar to the HVD (NPO capacitors and metallic resistors) and features a coaxial and compact structure in order to minimize parasitic effects and maintain excellent high-frequency behavior. The output of the attenuator is matched to the input impedance of the digitizer, typically also equal to 1 MΩ in parallel with 30 pF. Additionally, the time constant of the high-voltage part is matched with that of the low-voltage part to ensure good frequency behavior.

**Figure 4:**
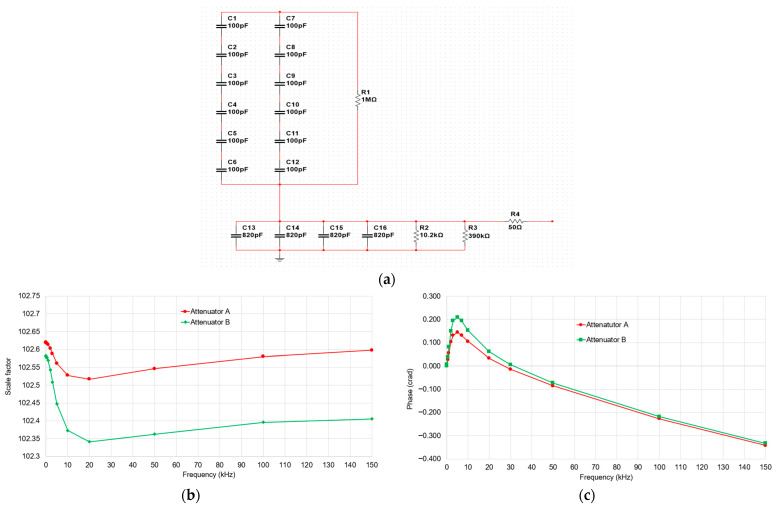
Developed attenuators. (**a**) Electrical circuit: (**b**) measurement results for amplitude response; (**c**) measurement results for phase response.

The combination of the HVD and the attenuator results in a total scale factor of 50,000. Consequently, when measuring a high-voltage signal of 50 kVp, the chain provides an output of 1 V, which can be accurately captured using the 1 V input range of the digitizer. Two attenuators have been developed: attenuator A is associated with the HVD, while attenuator B is potentially associated with the IT under calibration. Additional attenuators can be easily developed to match the scale factors of various ITs (e.g., attenuation of 5, 10, 50 and 200) or various impedances (e.g., 2 MΩ and 10 MΩ). The electrical circuit and the developed attenuators are shown in Figure 4.

From the results, it is shown that the frequency responses of both attenuators are relatively flat over the range from DC to 150 kHz. Attenuator A maintains a scale factor around 102.5, with minimal variation (~0.1%). Attenuator B shows slightly more variation, with a gradual decrease in the scale factor from approximately 102.6 to 102.3, which remains negligible. In terms of phase, the phase at the fundamental frequency is almost zero for both attenuators. A small overshoot is observed around 7 kHz; beyond that, the phase response is nearly linear, allowing for correction if necessary. Note that the scale factor and phase values are obtained under certain conditions; the load, specifically the input impedance of the digitizer, has a significant influence. For example, a 1 pF variation in the capacitance and 1% variation in the resistance of 1 MΩ affects the scale factor by about 0.01% and the phase by approximately 0.0015 crad. Therefore, the input impedance of the digitizer must be measured precisely to apply corrections if necessary.

### 3.3. Design of the Filter

The filter enhances the sensing capability for weak harmonics. The objective of the filter is to analyze high-frequency harmonic components with very small amplitudes (e.g., 0.01% of the fundamental). It is required to strongly attenuate the fundamental frequency (50 Hz) and its low-order harmonics, particularly up to a few hundreds of Hz. The goal is to suppress dominant low-frequency components and only retain harmonics in the range from 9 kHz to 150 kHz. To achieve sufficient rejection of the fundamental and its harmonics, a filter of at least the 4th order is necessary. The use of filters in harmonic measurement is common and has been the subject of numerous scientific studies [29,30,31]; we have decided to develop a high-pass filter (HPF) to analyze these suprahamonics.

The equivalent impedance of the filter needs to be high to not strongly disturb the metrological characteristics of the HVD. However, this high impedance comes with trade-offs: it tends to increase the cutoff frequency of the filter. In addition, the capacitor C6 of the filter needs to be much higher than the input capacitance of the digitizer (typically around 30 pF), because together they form a capacitive voltage divider. If the filter capacitor is too small, it will significantly reduce the signal reaching the digitizer, resulting in undesired attenuation of the output voltage. As a result, an ideal filter response is not achievable, and a balance must be found between attenuation performance and phase linearity; a small attenuation and phase shift will still be present in the range of 9 kHz–150 kHz.

Another limitation in the filter design arises from the input resistor of the digitizer, typically 1 MΩ, which is internally connected in parallel with its input capacitance. This resistor cannot be removed or modified, yet it forms part of the overall RC network seen by the filter. As a result, it introduces a fixed time constant that limits the ability to precisely control the cutoff frequency of the filter using the external resistor R7 alone. Even if R7 is carefully selected, the presence of the digitizer’s internal 1 MΩ resistor means that the effective resistance seen by the filter is not purely defined by R7, but by the parallel combination of R7 and the digitizer’s input resistance. This constraint reduces the flexibility in filter tuning and must be taken into account when designing the frequency response. In particular, it makes it more difficult to achieve sharp cutoffs or target specific bandwidths without compromising other aspects such as signal attenuation or phase linearity. A possible solution is to develop impedance matchers or active filters, but such developments will bring other difficulties such as electronic noise.

For these reasons, the filter is intended to be used only when the harmonic voltages are very low (typically < 0.1% of the fundamental), where precise filtering is critical. In cases where harmonics are more significant, the primary measurement chain (HVD + attenuator) is used without the filter to preserve accuracy. Because the implementation of the filter slightly modifies the electrical behavior of the measurement chain, particularly in terms of impedance, frequency response and phase behavior, it is essential to characterize the complete chain with the filter to ensure measurement reliability and traceability.

**Figure 5:**
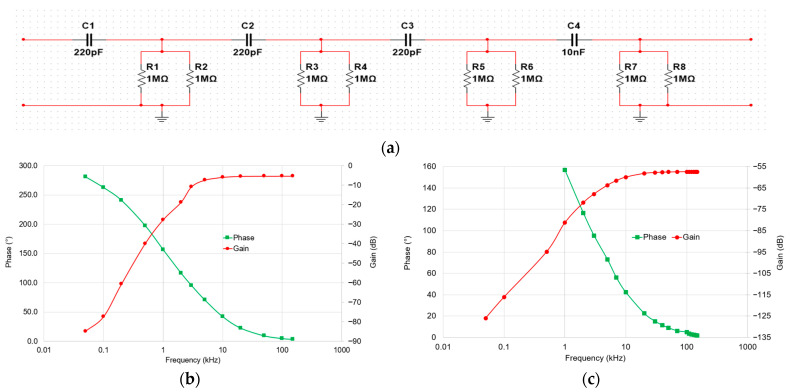
The developed filter: (**a**) electrical circuit; (**b**) gain and frequency results of the developed filter only; (**c**) gain and frequency results of the developed filter associated with the HVD.

We have chosen to set up a 4th-order low-pass filter using a cascade of RC (resistor–capacitor) stages. A 4th-order passive RC filter was selected to ensure stability, simplicity and reproducibility in the 9–150 kHz frequency range. Passive filters present the advantage of being inherently linear and free from additional noise or distortion that can be introduced by active components. A higher-order design was considered unnecessary as the 4th-order response already provided sufficient attenuation outside the band of interest while maintaining good phase characteristics. Each stage consisted of a 220 pF capacitor and a 500 kΩ resistor to ground, forming individual first-order low-pass filters. By cascading these stages, we obtained a sharper frequency roll-off characteristic of a 4th-order filter, which attenuates low-frequency components more aggressively. The capacitors and resistors were chosen from the same technologies as the HVD and the attenuator. The results of this filter are presented in Figure 5.

From the results of the filter alone (Figure 5b), it can be observed that the attenuation between 9 kHz and 150 kHz is approximately 5 dB. This attenuation is primarily due to the influence of the input impedance of the digitizer, as previously discussed. The 50 Hz fundamental component is attenuated by about 85 dB, while the 10th harmonic of the 50 Hz signal (i.e., 500 Hz) is attenuated by approximately 40 dB. When the filter is combined with the HVD (Figure 5c), the attenuation between 9 kHz and 150 kHz increases significantly, ranging from 60 dB to 57 dB, and the 50 Hz fundamental is attenuated by approximately 126 dB. These results demonstrate that the filter is sufficiently effective in suppressing both the fundamental and low-frequency harmonics. However, as previously noted, the impedance seen by the filter (e.g., impedance of the digitizer) has a strong influence on the gain and phase responses of the filter. According to the characterization results, a variation of just 1 pF in this impedance can lead to an error of approximately 0.9% in gain and 0.04 crad in phase at 150 kHz.

In practice, as explained in Section 5.4, the divider and associated filter are calibrated together as a complete chain to ensure traceability of the scale factor and frequency response in the 9–150 kHz range. Since the calibration is performed with an impedance slightly different from that of the digitizer input, a correction is applied during data processing to account for the exact input impedance of the digitizer. This guarantees that the influence of the filter and the impedance mismatch are both compensated for, ensuring accurate amplitude and phase measurements over the frequency range of interest.

## 4. Selection and Characterization of the Digitizer

### 4.1. Selection of the Digitizer

The digitizer completes the sensing chain by converting the conditioned analog signals into digital data for further analysis. To ensure accurate measurements, the digitizer used in the chain must meet several important requirements. Its input impedance, typically 1 MΩ//30 pF, needs to be known accurately in order to correct the measuring chains if needed in terms of both scale factor and phase. When used in combination with the filter, the digitizer characteristics become even more critical. In this configuration, the digitizer and filter together form a capacitive voltage divider, which can significantly affect the signal amplitude if not properly accounted for. Therefore, the input capacitance of the digitizer must be precisely known and considered during chain calibration. The corrections of the input impedances of the digitizers are usually obtained by experimentations.

The input voltage range must be compatible with the output of the measurement chain, ideally allowing a 1 Vp full-scale input when measuring up to 50 kVp, assuming a total scale factor of 50,000. The digitizer should provide high resolution, preferably 16 bits or more, to accurately capture small signal components such as harmonics down to 0.1% of the fundamental. A wide analog bandwidth is also essential; the digitizer should offer a bandwidth of at least several MHz to accurately capture signal content up to 150 kHz without amplitude distortion or phase error. A high sampling rate (e.g., 10 MS/s or higher) is required to prevent aliasing and enable precise time- and frequency-domain analysis.

Several digitizers were considered for high-voltage measurements, including the PicoScope 4262, Fluke 8588A, Keysight 3458A, NI PXIe-6396 and Applicos WFD22. While the Fluke and Keysight models offer excellent accuracy and resolution for low-frequency metrology, their single inputs necessitate the need to use two digitizers with a high-accuracy synchronization chain. The NI PXIe-6396 and Applicos WFD22 provide higher performance and resolution, but require more complex integration and software development. The PicoScope 4262 was ultimately chosen for its ideal balance of low noise (~8.5 µV RMS), 16-bit resolution, 5 MHz bandwidth and 10 MS/s sampling rate, along with fast USB communication, easy programming and two internally synchronized channels. The datasheet of the PicoScope 4262 does not specify the time delay between the two channels. This delay has been determined during the calibration of the instrument. An alternative method to mitigate a possible delay is to interchange the oscilloscope channels so that the effect cancels out. However, we chose to include this delay directly in the measurement uncertainty in Section 5. These features make it well suited for capturing small harmonic signals up to 150 kHz and for rapid integration.

### 4.2. Characterization of the Digitizer

**Figure 6:**
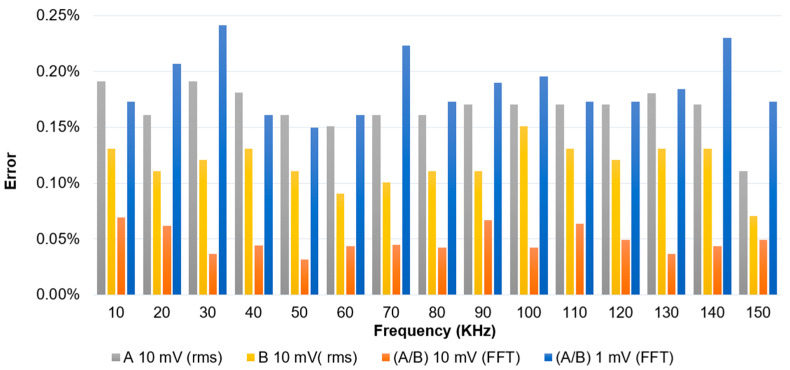
Digitizer error across a range of frequencies (10 kHz to 150 kHz) for different measurement methods (rms and FFT) and signal amplitudes (1 mV and 10 mV) in ± 1 V range.

The PicoScope 4262 was characterized in the ±1 V range by measuring small signals of 10 mV and 1 mV amplitude, corresponding to 1% and 0.1% of the range, respectively. This was performed to assess the scope’s ability to detect low-level signals within a high dynamic range in the frequency from 10 kHz to 150 kHz. The oscilloscope was configured identically to its operational setup for the acquisition of a fundamental @50 Hz and the superimposed harmonics: with a 5 MHz analog bandwidth, 10 MS/s sampling rate and horizontal resolution set to 20 ms/div. Measurements were carried out using two methods: direct time-domain acquisition with RMS readings and frequency-domain analysis using FFT. While the RMS method provides a general measure of signal energy, it incorporates all harmonic components, along with noise, offset and spurious signals, which reduces its accuracy, especially at low amplitudes around 0.1% of the range. In contrast, the FFT method is more representative, as it allows for direct extraction of specific harmonic amplitudes, making it more suitable for evaluating small-signal detection. The results were expressed either in terms of absolute amplitude or as the ratio of amplitudes and their phase shifts between two input channels. This ratio is particularly relevant, as it reflects the actual quantity being measured in the chain according to the governing Equations (1) and (2). Using the amplitude ratio also helps reduce sensitivity to absolute gain variations or noise, and thus provides a more accurate evaluation of the chain harmonic measurement performance. The results are presented in Figure 6.

It is evident that RMS-based measurements, particularly for individual channels (A and B), exhibit higher errors, around 0.2% at 10 mV. At the 1 mV level, the rms results are unusable due to the influence of noise, offset and non-harmonic components included in the rms calculation. The amplitude ratio (A/B) shows significantly lower and more stable errors, typically below 0.05%, demonstrating improved accuracy in detecting small harmonic components. Even at very low signal levels (1 mV or 0.1% of the range), the FFT method maintains acceptable performance, with errors remaining under 0.3% across the frequency range. In terms of phase shift between both inputs, an error of approximately 0.1 crad is observed up to 150 kHz, measured by applying the same signal to both inputs. At the fundamental frequency (AC or DC), the FFT technique has also been used to measure the amplitude ratio (A/B), with observed errors around 0.04% with an associated uncertainty of 0.01%.

These results confirm that the PicoScope 4262, when used with FFT-based ratio measurements, provides a more accurate and representative evaluation of harmonic components in high-dynamic-range conditions, particularly when signal amplitudes are low. For harmonic voltages lower than 0.1% of the range and down to 0.01%, the use of the dedicated filter becomes beneficial. In this case, the filter enhances the harmonic component while suppressing the fundamental, so that the voltage applied to the digitizer remains above 1 mV, making it measurable with acceptable error levels, typically around 0.3%, as demonstrated.

## 5. Estimation of Measurement Uncertainty

### 5.1. Measurement Procedures

According to Figure 2, two measurement paths are available. The first path, consisting of the HVD followed by the attenuator (scale factor of 50,000), is used to measure the fundamental frequency and any harmonic components with amplitudes greater than 0.1% of the fundamental. The maximum test voltage is 50 kV according to the characteristics of the HVD. In this case, 0.1% of the fundamental corresponds to 50 V at the divider input, i.e., 1 mV at the measurement output. However, if the test voltage is lower than 50 kV, the relative criterion of 0.1% is no longer meaningful. Therefore, there is a minimum limit of 1 mV at the output, regardless of the actual test voltage.

The second path, which bypasses the attenuator and uses the HPF, is configured to only measure harmonics with amplitudes as low as 0.01% of the fundamental (or output voltage < 1 mV if using the first path). Since this path avoids additional attenuation, it offers a better signal-to-noise ratio for detecting weak harmonics that might otherwise be buried in the noise floor or undetectable due to limited resolution.

This separation of signal acquisition paths ensures the following:

- Path 1: (HVD + attenuator) for the measurement of fundamental and harmonic amplitudes as low as 0.1% of the fundamental (or output voltage as low as 1 mV);
- Path 2: (HVD + HPF) for the measurement of weak harmonics as low as 0.01% of the fundamental (or output voltage lower than 1 mV using the first path).

The calibration uncertainty depends solely on the calibration uncertainty of the reference measuring chain (Path 1 or Path 2) and on the voltage ratio measured at the digitizer side. The influence of temperature has been neglected, since all components used in the development have very small temperature coefficients, and all measurements are performed in a high-precision, temperature-controlled room at (23 ± 1) °C.

### 5.2. Uncertainty Estimation for the Fundamental Component

The main uncertainty contributions for the measurement of the fundamental are as follows:

- Calibration of Path 1: To achieve low uncertainty at the fundamental, the entirety of Path 1 is calibrated using our internal reference procedure that accounts for stray impedance effects. Calibration is performed with an impedance equivalent to that of the digitizer. The resulting standard uncertainty is 0.0015% for amplitude (AC and DC) and 0.0015 crad for phase.
- Digitizer calibration: The voltage ratio is calibrated in both amplitude and phase by injecting two known voltage signals directly into the digitizer inputs in the same configuration as it is used during high-voltage measurements. The signals are analyzed using FFT to determine the complex ratio between channels. The estimated standard uncertainties are 0.01% for amplitude (AC and DC) and 0.01 crad for phase (at 50 Hz).
- Influence of the attenuator load: The uncertainty caused by load effects is included in the overall uncertainty budget since Path 1 is calibrated without the digitizer. The input impedance of the digitizer needs to be accurately determined to correct this influence if needed. We consider an associated uncertainty of 1 pF for the capacitive part and 1% for the resistive part, resulting in an estimated uncertainty of 0.01% for amplitude (AC and DC) and approximately 0.0015 crad for phase—considered negligible.

### 5.3. Uncertainty Estimation for Harmonics Down to 0.1% of the Fundamental

To ensure accurate harmonic analysis from 9 kHz to 150 kHz, we set a threshold at 0.1% of the fundamental (or output voltage > 1 mV if using Path 1). Harmonic components above this threshold are acquired using Path 1. To maintain the required measurement uncertainty, each component of Path 1 (HVD, attenuator) must be calibrated separately. Calibrating the entire path as a whole can degrade accuracy, especially for phase, due to the small voltage on the output of Path 1 (scale factor of 50,000). Calibrating each part independently under controlled conditions minimizes uncertainty and ensures traceability. The main contributions are as follows:

- HVD Calibration: Calibrated up to 150 kHz. From Table 1, a standard uncertainty of 0.1% is adopted for amplitude. For phase, a correction factor of 38 ns is applied, with a standard uncertainty of 1 ns (equivalent to 0.1 crad at 150 kHz). The HVD is calibrated with a load equivalent to the attenuator (30 pF//1 MΩ).
- HVD Load Sensitivity: A variation of 1 pF in capacitance and 1% in the 1 MΩ resistive load introduces a standard uncertainty of approximately 0.005% for the amplitude and a negligible phase uncertainty over 9 kHz to 150 kHz.
- Attenuator Calibration: Calibrated up to 150 kHz in both amplitude and phase. From Figure 4, a standard uncertainty of 0.1% is adopted for amplitude. For phase, a correction is applied (Figure 4c), with a standard uncertainty of 0.1 crad.
- Attenuator Load Sensitivity: A 1 pF change in capacitance and 1% variation in resistive load introduces an uncertainty of approximately 0.005 crad in phase and 0.03% in gain over the 9 kHz to 150 kHz range.
- Digitizer Calibration: As shown in Figure 6, for harmonic voltages at 0.1% of the fundamental, a standard uncertainty of 0.3% can be adopted. For 1% of the fundamental, 0.1% uncertainty is applicable. The associated phase uncertainty is 0.1 crad.

### 5.4. Uncertainty Estimation for Harmonics Down to 0.01% of the Fundamental

For accurate detection of very-low-level harmonics down to 0.01% of the fundamental (or output voltage < 0.1 mV if using the path1), we use Path 2 (HVD + Filter). The output voltage of Path 2 is then higher than 1 mV due to its low attenuation (about 60 dB, as presented in Figure 5c).

The uncertainty contributions come primarily from Path 2 and the digitizer.

- Path 2 Calibration: To achieve accurate results, Path 2 can be calibrated at 20 V, from 9 kHz to 150 kHz, using our reference procedure that accounts for stray impedance effects. Calibration is performed with a load equivalent to that of the digitizer. The resulting standard uncertainties are 0.2% for amplitude and 0.2 crad for phase.
- Digitizer Calibration: As described earlier, the voltage ratio is calibrated using two known signals and FFT analysis. Given the scale factor of Path 2 (up to 3000), a harmonic at 0.01% of a 5 V fundamental results in an up to 1.6 mV signal at the digitizer input. This can be easily acquired using the 10 mV range of the digitizer. The estimated standard uncertainty is 0.2% for amplitude and 0.1 crad for phase.
- Filter Load Sensitivity: A 1 pF variation in capacitance and 1% in the 1 MΩ resistive load introduce a phase uncertainty of approximately 0.04 crad and a gain uncertainty of about 0.9% in the 9 kHz to 150 kHz frequency range.

The calibration uncertainty of the measurement chain was evaluated across the frequency range of 9 kHz to 150 kHz using the GUM [32]. The results are summarized in Table 2 and Table 3, respectively, for amplitude and phase. The expanded uncertainties (with a coverage factor k ≈ 2) were determined for different amplitude levels of harmonic components relative to the fundamental. Equation (4) has been used for the calculation of the expanded uncertainty.

(4) $$U=2\;\sqrt{\sum u_{i}^{2}}$$

$u_{i}$ is the standard uncertainty of each contribution source.

**Table 2:** Calibration uncertainty of instrument transformers for amplitude.

Contribution/Source	Fundamental (Path 1)	Harmonics > 0.1% Output Voltage > 1 mV (Path 1)	Harmonics > 0.01% Output Voltage > 1 mV (Path 2)
Calibration of path/HVD	0.0015%	0.1%	0.2%
Digitizer calibration	0.01%	0.1% (at 1% fund.) 0.3% (at 0.1% fund.)	0.2%
Attenuator calibration	–(included in path)	0.1%	-
Load sensitivity	0.01% (attenuator)	0.005% (HVD) 0.03% (attenuator)	0.9% (filter)
Expanded uncertainty (k = 2)	0.028%	0.29% (at 1% fund.) 0.45% (at 0.1% fund.)	1.9% (at 0.01% fund.)

**Table 3:** Calibration uncertainty of instrument transformers for phase.

Contribution/Source	Fundamental (Path 1)	Harmonics > 0.1% Output Voltage > 1 mV (Path 1)	Harmonics > 0.01% Output Voltage > 1 mV (Path 2)
Calibration of path/HVD	0.0015 card	0.1 card	0.2 crad
Digitizer calibration	0.01 card	0.1 crad	0.1 crad
Attenuator calibration	–(included in path)	0.1 crad	-
Load sensitivity	0.005 card (attenuator)	0.005% (HVD) 0.005 crad (attenuator)	0.04 crad (filter)
Expanded uncertainty (k = 2)	0.020 crad	0.3 crad	0.5 crad

The results presented in Table 2 show that the measurement chain achieves expanded uncertainties close to the target values that we have fixed and required for the conformity assessment of ITs according to IEC 61869 Class WB3. For the fundamental frequency, the uncertainty is slightly above the ideal target (0.028% vs. 0.02%), but remains acceptable for most practical applications. For harmonics with voltage output higher than 0.1%; the uncertainties (0.29% and 0.45%, respectively) are also just above the target thresholds (0.2% and 0.4%), yet still demonstrate adequate performance for PQ evaluation in the 9 kHz to 150 kHz range. For very-low-level harmonics, 0.01% of the fundamental, the expanded uncertainty reaches 2%, which is two times higher than the fixed target uncertainty (1% up to 150 kHz). The major contribution here is the load sensitivity of the filter, which can be improved as explained in the Conclusion.

## 6. Discussion

The results presented in Section 5 confirm the ability of the proposed chain to achieve measurement uncertainties compatible with the requirements of IEC 61869 Class WB3. At the fundamental frequency, the expanded uncertainty is 0.028%, which is slightly above the initial target (0.02%) but still sufficient to support metrological Class 0.1 conformity assessments. For harmonic components down to 0.1% of the fundamental, the uncertainties remain below 0.5%, while for very-low-level harmonics (0.01% of the fundamental), the expanded uncertainty reaches 1.9%. Although this value is higher than the 1% target, it remains well within the 5% maximum permissible error allowed by the standard. These findings demonstrate that the chain provides a reliable tool for the traceable calibration and conformity assessment of medium-voltage ITs in the frequency range up to 150 kHz. The dual-path architecture ensures flexibility: Path 1 (HVD + attenuator) offers accurate characterization of fundamental and dominant harmonics, while Path 2 (HVD + HPF) extends sensitivity to weak harmonic components that would otherwise remain undetectable. This hybrid approach ensures both robustness and adaptability.

Nevertheless, the analysis also highlights the main limitation of the chain: the sensitivity of the filter to load variations. A variation of only 1 pF in the digitizer input capacitance produces an error close to 1% in amplitude at 150 kHz, which becomes the dominant source of uncertainty for very-low-level harmonics. To address this limitation, future improvements could include the implementation of an impedance matching stage, such as a unity-gain buffer amplifier. This buffer must feature extremely low noise and be dedicated to extremely low-voltage measurement. Such an approach would minimize the influence of the digitizer input impedance, currently the major contributor to measurement uncertainty, thereby enhancing accuracy for very-low-amplitude harmonics. Designing a buffer stage to measure signals in the millivolt range up to 150 kHz presents a fundamental trade-off between low noise and high bandwidth.

Similar works are currently in progress at other National Metrology Institutes. At RISE (Sweden), a reference chain is being developed based on a voltage divider with a scale factor of 26,000 and dual outputs, similar in concept to our dual-path architecture. The first output can be directly connected to a digitizer, while the second output is processed through a high-pass filter followed by a selectable-gain amplifier (gains of 10, 20 and 100). The amplified signals are acquired using a high-resolution digitizer (PXIe-6396, eight simultaneous inputs, 18-bit resolution, ≤15 MS/s and 1 MHz bandwidth). This chain is still under development and is expected to be reported in a future publication.

At VTT (Finland), another chain is being developed, also based on a high-voltage divider with a scale factor of 2000 that can be followed by an attenuator to extend the scale factor to 200,000. The wideband signal is digitized using the Applicos WFD20 card, which features 20-bit resolution, a maximum sampling rate of 2 MS/s, a 2 MHz (–3 dB) bandwidth and a 108 dB spurious-free dynamic range. The digitizer has two simultaneously sampling channels, allowing the calibration of both the magnitude and phase errors of the measuring chain connected in parallel with the reference divider.

Once these chains are fully characterized, an international comparison between our chain, the RISE chain and the VTT chain is foreseen. This comparison will validate the uncertainty budgets of the different approaches and will be the subject of a future joint publication.

Compared to existing chains, the proposed solution represents a significant advancement. The EMPIR IT4PQ project [16] addressed IT calibration for power quality but was limited to 9 kHz and harmonics higher than 1% of the fundamental, thus not covering the supraharmonic ranges, especially the weak ones. In [17,18], simplified methods were developed which, although suitable for industrial contexts, were restricted to 20 kHz, did not achieve uncertainties compatible with Class WB3 requirements and were unable to address weak harmonics. More recently, [19] considered realistic operating conditions but again did not extend measurements beyond 20 kHz and did not focus on weak harmonics.

By contrast, the chain presented in this work is the first to extend a reference architecture to 150 kHz with demonstrated metrological traceability even for weak harmonics. Its uncertainties, particularly for harmonics above 0.1% of the fundamental, are lower than those previously reported in the literature. A comparative summary of the main approaches is presented in Table 4.

The results obtained within this work, as well as those under development at other NMIs, are of direct relevance to ongoing standardization activities within IEC TC 38 (instrument transformers). Depending on the final outcomes from different reference chains, recommendations may be provided to the committee regarding the requirements defined in IEC 61869-1.

At LNE, the uncertainties achieved for the fundamental and for harmonics down to 0.1% of the fundamental remain significantly lower than the limits specified in IEC 61869-1, confirming that the reference method is well suited for conformity assessment. However, when the harmonic content reaches very low levels (0.01% of the fundamental), the expanded uncertainty (1.9%) of our chain approaches the maximum tolerance specified in the standard (5%). This shows that, while traceable measurements are still feasible, the margin between achievable uncertainties and normative requirements becomes very narrow at these low harmonic levels.

It is important to note that the uncertainties achieved at NMIs represent the best attainable under highly controlled laboratory conditions. In industrial practice, particularly for very low harmonic levels, it is likely that the tolerances declared in the standard will be extremely difficult to meet. This further justifies the need for clear recommendations to TC 38, distinguishing between the requirements applicable to reference laboratories and those realistically achievable in industrial environments.

Once the results from the other partners, in particular RISE and VTT, are available, a more consolidated set of recommendations can be formulated. These will include clarifications on how to distinguish the conformity requirements depending on the level of harmonic components superimposed on the fundamental, and how to set realistic requirement targets for manufacturers and testing laboratories. This coordinated effort will strengthen the basis for future revisions of IEC 61869-1 and support the definition of accuracy classes that are both technically achievable and aligned with metrological best practices.

## 7. Conclusions

A reference measurement chain for the assessment of medium-voltage ITs in the extended frequency range up to 150 kHz has been developed and validated. The chain is based on a dual-path architecture combining a wideband high-voltage divider, precision attenuators and a dedicated high-pass filter, enabling both accurate fundamental measurements and the detection of very-low-level harmonic components.

The results demonstrate that the chain achieves expanded uncertainties of 0.028% at the fundamental frequency, below 0.5% for harmonic components down to 0.1% of the fundamental and around 1.9% for very-low-level harmonics at 0.01% of the fundamental. Although these latter results remain compatible with the IEC 61869 Class WB3 requirements, the margin becomes narrow at such low levels. This limitation is mainly due to the sensitivity of the high-pass filter to load conditions and further improvements in the filter design are expected to reduce the uncertainty below the current 1.9% and extend the chain to the WB4 range up to 500 kHz.

An international comparison is planned with the reference chains currently under development at RISE and VTT. This will serve to consolidate the uncertainty evaluations and will provide a robust basis for recommendations to the IEC TC 38 committee in order to better define the requirements of the standard by distinguishing between what can be achieved at NMIs and what is realistically attainable by transformer manufacturers and testing laboratories.

Finally, the chain described in this work can be reproduced by transformer manufacturers and adapted either for direct use in grid measurements under realistic conditions or for the characterization of their products in test laboratories. This makes it not only a reference for NMIs but also a practical tool for industry. This establishes the chain not only as a reference measurement setup but also as a high-voltage sensor platform that can be reproduced and adapted for industrial sensing applications.

## Figures and Tables

**Table 1:** Calibration results of the HVD.

Condition	Voltage Range	Scale Factor	Relative Deviation	Remarks
DC	1 kV to 50 kV	500.848	0.004%	Voltage response
AC/50 Hz	1 kV to 35 kV rms	500.729	0.011%	Voltage response
DC to 150 kHz	200 V	500.37	0.07%	Frequency response
Phase shift	200 V			Time constant = −38 ns

**Table 4:** Comparison of existing chains and the proposed reference chain.

System	Frequency	Measurement Uncertainty	Architecture	Limitations
[16]	up to 9 kHz	[0.05% to 0.1%]	Reference setup Divider + digitizer	No coverage above 9 kHz Not WB3 compliant Not for weak harmonics
[17,18]	up to 20 kHz	1%	Simplified setup	Not WB3 compliant Not for weak harmonics
[19]	up to 20 kHz	[0.5–1]%	Realistic conditions	No extension to 150 kHz Not for weak harmonics
This work	up to 150 kHz	0.028% (fund.) 0.29% (at 1% fund.) 0.45% (at 0.1% fund.)	Dual-path RHVC: HVD + attenuator + HPF + digitizer	Sensitive to filter load but could be improved

## Data Availability

The original contributions presented in this study are included in the article. Further inquiries can be directed to the corresponding author.

## References

[1] Rönnberg S.K., Bollen M.H.J., Amaris H., Chang G.W., Gu I.Y., Kocewiak H., Meyer J., Olofsson M., Ribeiro P.F., Desmet J. (2017). On waveform distortion in the frequency range of 2 kHz–150 kHz—Review and research challenges. Electr. Power Syst. Res..

[2] Thomas T., Michael P.A. (2020). A review of high-frequency emission in 2–150 kHz range. Int. J. Adv. Appl. Sci..

[3] Agazar M., D’Avanzo G., Frigo G., Giordano D., Iodice C., Letizia P.S., Luiso M., Mariscotti A., Mingotti A., Muñoz F. (2024). Power grids and instrument transformers up to 150 kHz: A review of literature and standards. Sensors.

[4] Rönnberg S.K., Bollen M.H.J. (2016). Power quality issues in the electric power system of the future. Electr. J..

[5] Letha S.S., Espin Delgado A., Rönnberg S.K., Bollen M.H.J. (2021). Evaluation of medium-voltage networks for propagation of supraharmonic resonance. Energies.

[6] Novitskiy A., Schlegel S., Westermann D. Measurements and Analysis of Supraharmonic Influences in a MV/LV Network Containing Renewable Energy Sources. Proceedings of the 2019 Electric Power Quality and Supply Reliability Conference (PQ) & 2019 Symposium on Electrical Engineering and Mechatronics (SEEM).

[7] (2002). Electromagnetic Compatibility (EMC)—Part 4–7: Testing and Measurement Techniques—General Guide on Harmonics and Interharmonics Measurements and Instrumentation for Power Supply Systems and Equipment Connected Thereto.

[8] (2008). Electromagnetic Compatibility (EMC)—Part 4–30: Testing and Measurement Techniques—Power Quality Measurement Methods.

[9] Mendes T.M., Duque C.A., da Silva L.R.M., Ferreira D.D., Meyer J., Ribeiro P.F. (2020). Comparative analysis of measurement methods for the supraharmonic range. Int. J. Electr. Power Energy Syst..

[10] Istrate D., Amaripadath D., Toutain E., Roche R., Gao F. (2020). Traceable measurements of harmonic (2 to 150) kHz emissions in smart grids: Uncertainty calculation. J. Sens. Sens. Syst..

[11] Amaripadath D., Roche R., Joseph-Auguste L., Istrate D., Fortuné D., Braun J.P., Gao F. Measurement and analysis of supraharmonics emissions in smart grids. Proceedings of the 54th International Universities Power Engineering Conference (UPEC).

[12] Stano E., Kaczmarek P., Kaczmarek M. (2022). Why Should We Test the Wideband Transformation Accuracy of Inductive Current Transformers?. Energies.

[13] (2023). Instrument Transformers—Part 1: General Requirements.

[14] (2016). Instrument Transformers—Part 6: Additional General Requirements for Low-Power Instrument Transformers.

[15] (2017). General Requirements for the Competence of Testing and Calibration Laboratories.

[16] European Association of National Metrology Institutes EMPIR 19NRM05 IT4PQ Project Website. https://www.euramet.org/seg-it4pq1.

[17] Crotti G., Giordano D., D’Avanzo G., Letizia P.S., Luiso M. (2021). A new industry-oriented technique for the wideband characterization of voltage transformers. Measurement.

[18] Mingotti A., Betti C., Peretto L., Tinarelli R. (2022). Simplified and low-cost characterization of medium-voltage low-power voltage transformers in the power-quality frequency range. Sensors.

[19] Letizia P.S., Crotti G., Mingotti A., Tinarelli R., Chen Y., Mohns E., Agazar M., Istrate D., Ayhan B., Çayci H. (2023). Characterization of instrument transformers under realistic conditions: Impact of single and combined influence quantities on their wideband behavior. Sensors.

[20] Stano E., Kaczmarek P., Kaczmarek M. (2023). Evaluation of the optional wideband accuracy of inductive current transformers in accordance with IEC 61869-1 Ed. 2. Energies.

[21] Giordano D., Crotti G., D’Avanzo G., Delle Femine A., Iodice C., Luiso M., Letizia P.S. Characterizing voltage transformers up to 150 kHz: A new approach to generate MV distorted test waveforms. Proceedings of the Conference on Precision Electromagnetic Measurements (CPEM).

[22] Chen Y., Mohns E., Badura H., Räther P., Luiso M. Setup and characterisation of reference current-to-voltage transformers for wideband CT calibration up to 2 kA. Proceedings of the IEEE AMPS Workshop.

[23] Buchhagen C., Fischer M., Hofmann L., Däumling H. Metrological Determination of the Frequency Response of Inductive Voltage Transformers up to 20 kHz. Proceedings of the 2013 IEEE Power & Energy Society General Meeting.

[24] Pogliano U., Trinchera B., Serazio D. (2012). Wideband Digital Phase Comparator for High Current Shunts. Metrologia.

[25] Yan W., Emms F., Dewayalage I., Li Y., Budovsky I. A reference measurement system for calibration of high-voltage transducers at frequencies up to 10 kHz. Proceedings of the IEEE Workshop on Applied Measurements for Power Systems (AMPS).

[26] Rom M., van den Brom H.E., Houtzager E., van Leeuwen R., van der Born D., Rietveld G., Muñoz F. (2025). Measurement system for current-transformer calibration from 50 Hz to 150 kHz using a wideband power analyzer. Sensors.

[27] European Association of National Metrology Institutes EPM 22NRM09 ADMIT Project Website. https://www.admit-project.eu/.

[28] Agazar M., Saadeddine H., Dougdag K., Ouameur M., Azzoug M. (2025). The Design and Development of a Low-Cost and Environmentally Friendly Voltage Divider for On-Site High-Voltage Calibration up to 850 kV. Sensors.

[29] Mendes T.M., Duque C.A., Silva L.R.M., Ferreira D.D., Meyer J. (2019). Supraharmonic analysis by filter bank and compressive sensing. Electr. Power Syst. Res..

[30] Morán L., Dixon J., Torres M., Rashid M.H. (2018). 41—Active Power Filters. Power Electronics Handbook.

[31] Bagheri P., Xu W., Ding T. (2016). A Distributed Filtering Scheme to Mitigate Harmonics in Residential Distribution Systems. IEEE Trans. Power Deliv..

[32] (2008). Uncertainty of Measurement—Part 3: Guide to the Expression of Uncertainty in Measurement (GUM: 1995).

